# Alterations of the genes involved in the PI3K and estrogen-receptor pathways influence outcome in human epidermal growth factor receptor 2-positive and hormone receptor-positive breast cancer patients treated with trastuzumab-containing neoadjuvant chemotherapy

**DOI:** 10.1186/1471-2407-13-241

**Published:** 2013-05-16

**Authors:** Mamoru Takada, Toru Higuchi, Katsunori Tozuka, Hiroyuki Takei, Masayuki Haruta, Junko Watanabe, Fumio Kasai, Kenichi Inoue, Masafumi Kurosumi, Masaru Miyazaki, Aiko Sato-Otsubo, Seishi Ogawa, Yasuhiko Kaneko

**Affiliations:** 1 Department of Cancer Diagnosis, Research Institute for Clinical Oncology, Saitama Cancer Center, 818 Komuro, Ina, Saitama, 362-0806, Japan; 2 Department of General Surgery, Graduate School of Medicine, Chiba University, Chiba, Japan; 3 Divisions of Breast Surgery, Saitama Cancer Center, Ina, Saitama, Japan; 4 Divisions of Breast Oncology, Saitama Cancer Center, Ina, Saitama, Japan; 5 Department of Pathology, Saitama Cancer Center, Ina, Saitama, Japan; 6 Cancer Genomics Project, Graduate School of Medicine, University of Tokyo, Tokyo, Japan

**Keywords:** HER2, SNP array, Trastuzumab, Neoadjuvant chemotherapy, PI3K pathway, Estrogen receptor pathway, Complete pathological response, Relapse-free survival

## Abstract

**Background:**

Chemotherapy with trastuzumab is widely used for patients with human epidermal growth factor receptor 2-positive (HER2+) breast cancer, but a significant number of patients with the tumor fail to respond, or relapse. The mechanisms of recurrence and biomarkers that indicate the response to the chemotherapy and outcome are not fully investigated.

**Methods:**

Genomic alterations were analyzed using single-nucleotide polymorphism arrays in 46 HER2 immunohistochemistry (IHC) 3+ or 2+/fluorescent in situ hybridization (FISH)+ breast cancers that were treated with neoadjuvant chemotherapy with paclitaxel, cyclophosphamid, epirubicin, fluorouracil, and trastuzumab. Patients were classified into two groups based on presence or absence of alterations of 65 cancer-associated genes, and the two groups were further classified into four groups based on genomic *HER2* copy numbers or hormone receptor status (HR+/−). Pathological complete response (pCR) and relapse-free survival (RFS) rates were compared between any two of the groups.

**Results and discussion:**

The pCR rate was 54% in 37 patients, and the RFS rate at 3 years was 72% (95% CI, 0.55-0.89) in 42 patients. The analysis disclosed 8 tumors with nonamplified *HER2* and 38 tumors with *HER2* amplification, indicating the presence of discordance in tumors diagnosed using current HER2 testing. The 8 patients showed more difficulty in achieving pCR (*P*=0.019), more frequent relapse (*P*=0.018), and more frequent alterations of genes in the PI3K pathway (*P*=0.009) than the patients with *HER2* amplification. The alterations of the PI3K and estrogen receptor (ER) pathway genes generally indicated worse RFS rates. The prognostic significance of the alterations was shown in patients with a HR+ tumor, but not in patients with a HR- tumor when divided. Alterations of the PI3K and ER pathway genes found in patients with a HR+ tumor with poor outcome suggested that crosstalk between the two pathways may be involved in resistance to the current chemotherapy with trastuzumab.

**Conclusions:**

We recommend FISH analysis as a primary HER2 testing because patients with IHC 2+/3+ and nonamplified *HER2* had poor outcome. We also support concurrent use of trastuzumab, lapatinib, and cytotoxic and anti-hormonal agents for patients having HR+ tumors with alterations of the PI3K and ER pathway genes.

## Background

Patients with human epidermal growth factor receptor 2 (HER2)-positive (HER2+) breast cancer were known to have a poor prognosis in the era when trastuzumab was not available [1-3]. After the introduction of trastuzumab, the outcome of HER2+ operable patients changed significantly, and many patients who achieved a pathological complete response (pCR) were expected to have been cured of the disease [2,3]. However, pCR rates are 30-60%, and the 3-year relapse-free survival (RFS) is 71-78% in patients with operable breast cancer, indicating that a substantial number of patients who undergo surgical resection after the chemotherapy have recurrence [2,3]. Patients with HER2+ breast cancer are usually treated with a combination of trastuzumab and taxanes with or without other chemotherapeutic agents [1-3], but predictors that indicate the response to the chemotherapy and outcome are not fully investigated.

Alterations in the HER2-PI3K-AKT pathway, which include expression of an extracellular domain-truncated HER2 (p95HER2), mutation and amplification of *PIK3CA*, loss of *PTEN* or *INPPB4*, and mutation of *AKT1*, are known to result in a poor response to chemotherapy with trastuzumab or poor outcome for breast cancer patients [4,5]. In addition, there are two types of HER2+ breast cancer; namely, hormone receptor (HR)-positive (HR+) and HR-negative (HR-), and some investigators have reported different biological characteristics including pathological responses between the two [6,7]. Crosstalk between the estrogen receptor (ER) pathway and the PI3K or ERK/MAPK pathway is thought to be involved in the resistance to trastuzumab-containing chemotherapy in HER2+/HR+ breast cancer [8]. However, there have been few studies aiming to resolve the mechanism of chemotherapy resistance or to identify biomarkers that indicate pCR and relapse using clinical samples.

It has been reported that 0.9% - 18.5% of HER2 immunohistochemistry (IHC) 3+ tumors had a single copy of *HER2*[8]. The technical shortcomings of IHC that can result in false-positive and false-negative results may be one of the reasons for the discordance between IHC grades and *HER2* copy numbers [9,10], however, there may be true single-gene overexpressers although the incidence may be low. Although metastatic breast cancer patients with the discordance between IHC and *HER2* copy numbers seemed to show a low probability of responding to HER2-targeted therapy [11], there has been no study to clarify that single-gene overexpressers with operable breast cancer will respond to trastuzumab, and the mechanisms for the possible resistance to the trastuzumab-containing chemotherapy.

An alternatively spliced form of the human *HER2* gene, Δ16*HER2*, containing an in-frame deletion was found in human breast cancer [12]. Mitra et al. showed that ectopic expression of the Δ16*HER2* transcript, but not wild-type *HER2* transcript, promotes receptor dimerization, cell invasion, and trastuzumab resistance in NIH3T3 and MCF7 tumor cells [13]. More recently, it was reported that Δ16*HER2*-expressing transgenic mice, but not wild-type *HER2*-expressing mice, developed multiple mammary adenocarcinomas [14]. However, the clinical significance of Δ16*HER2* has not been fully examined in human breast cancer.

We hypothesized that genomic alterations detectable by single-nucleotide polymorphism (SNP) arrays and *HER2* copy numbers and levels of *HER2* transcripts would suggest mechanisms of resistance and prognostic factors for patients treated with trastuzumab-containing chemotherapy. Thus, we studied SNP array patterns of 143 breast cancer samples, including 46 HER2+ tumors, obtained at the time of diagnosis. We found that alterations of genes involved in the estrogen-receptor (ER) and PI3K pathways indicated worse RFS rates in patients with a HR+ but not HR- tumor, who were treated with chemotherapy with trastuzumab, followed by adjuvant trastuzumab (plus endocrine therapy for patients with a HR+ tumor). We also found that patients with a tumor showing a single *HER2* copy number had more difficulty in achieving pCR, and tended to have worse RFS rates than those having a tumor with *HER2* amplification. These findings may help to clarify the mechanisms for resistance to the chemotherapy with trastuzumab, and improve the efficacy of chemotherapy in HER2+ breast cancer.

## Methods

### Patients and samples

One hundred and fifty four tumor tissue and peripheral blood samples were obtained from 152 Japanese women, including two with bilateral tumors, who underwent a diagnostic core-needle biopsy between April 2005 and August 2011. The first specimen was used for the pathological diagnosis with H&E staining, the determination of ER, progesterone receptor (PgR), and HER2 status using IHC, and fluorescent in situ hybridization (FISH) [15]. The second and third specimens, which were directly frozen in liquid nitrogen, were used for DNA analysis including SNP assays and for RNA and definitive FISH analyses, respectively. Eleven specimens were excluded after evaluation of the content of tumor area, which was less than 30% of the whole specimen. Thus, 143 specimens from 141 patients were used for the present study. All patients included in the analysis provided consent to participate in the study and to publish the results. The study design was approved by the ethics committee of Saitama Cancer Center.

### Histological examination and immunohistochemistry

The core-needle specimens were evaluated microscopically by pathologists, and classified according to the system proposed by Elston and Ellis [16]. Positive rates (%) for the ER and PgR were determined as a ratio of positive cells to total cancer cells, and a value of 10% or higher was defined as positive [17]. HER2 expression was defined as 0 to 3+ based on positive cell rates and the intensity of IHC staining (HercepTest, DAKO, Japan). Tumors showing moderate expression (2+) of HER2 were also tested by FISH to clarify amplification of the *HER2* gene in paraffin specimens with the use of PathVysion (Abbott, IL); positive FISH was defined as a ratio of *HER2* signals to centromere 17 signals of >2.2. Thus, a HER2-positive reaction was defined as either 3+ for IHC or 2+ for IHC with positive routine FISH results.

P-cadherin (monoclonal mouse anti-human P-cadherin clone 56, BD Transduction Lab.) was subjected to immunohistochemical staining using an avidin-biotin complex for the validation of genomic alterations identified by the SNP array in breast cancers.

### Neoadjuvant and adjuvant chemotherapy and adjuvant hormone therapy

Of 141 patients, 46 were determined as having a HER2+ tumor. Of these 46 patients, 37 received neoadjuvant chemotherapy consisting of 12 weekly cycles of paclitaxel with trastuzumab, and four cycles of cyclophosphamide, epirubicin, and fluorouracil with concurrent trastuzumab throughout the chemotherapy [1,18], and then underwent surgery. Of the remaining 9 patients, 4 chose immediate surgery, one received neoadjuvant chemotherapy without trastuzumab, which was added after surgery, and three with metastatic cancers at diagnosis and one with bilateral tumors who chose treatment with trastuzumab, exemestane, and radiation did not undergo surgery. All 5 patients who underwent surgery, received essentially the same trastuzumab-containing chemotherapy, and were included in the RFS analysis. Thus, pathological response after neoadjuvant chemotherapy was evaluated in 37 tumors, and RFS was evaluated for 42 patients. After surgery, weekly trastuzumab therapy was given to HR- patients for 6 to 12 months [3], and the same therapy plus hormone therapy; tamoxifen for premenopausal patients and an aromatase inhibitor for postmenopausal patients, was given to patients with a HR+ tumor.

### Pathological response

Pathological response was assessed by a pathologist (M. K.) according to the “histopathological criteria for assessment of therapeutic response in breast cancer” proposed by the Japanese Breast Cancer Society [19]. The extent of responses is classified as grade 0, 1, 2, and 3, which represents no response, slight response, marked response, and complete response (CR), respectively. The grade 1 is further classified as 1a and 1b, which represents mild response indicating mild changes in cancer cells regardless of the area, or marked changes in less than one third of cancer cells, and moderate response indicating marked changes in one third or more but less than two thirds of tumor cells, respectively. Grade 2 indicates marked changes in two thirds or more of tumor cells. Grade 3 indicates necrosis or disappearance of all tumor cells.

### Copy number and loss of heterozygosity (LOH) analysis using SNP arrays

Affymetrix Mapping 250K-Nsp arrays (Affymetrix, Santa Clara, CA) were used to analyze the chromosomal copy number and LOH status in 143 tumors as described previously [20]. Partial uniparental disomy (UPD) was defined as a region of copy number-neutral LOH spanning over 3 Mb. Copy numbers and LOH were calculated using CNAG and AsCNAR programs with paired references as controls [21,22]. Amplification, gain, and loss are defined as copy number ratios of >2.5, 1.2-2.5, and <0.8, respectively.

Sixty-five genes were chosen for analyzing genomic alterations (gain, amplification, loss, and UPD) because of previous studies reporting the involvement of these genes in the neoplastic process of breast and other cancers. They were *GPSM2*, *GSTM1*, *ATR*, *PIK3CA*, *MUC4*, *INPP4B*, *TERT*, *MAP3K1*, *CCNB1*, *GCCR*, *FOXC1*, *DEK*, *ID4*, *E2F3*, *NOTCH4*, *VEGFA*, *ESR1*, *HOXA9*, *CHIP*, *MET*, *EGR3*, *FGFR1*, *MYC*, *CDKN2A*, *BAG1*, *CTSL2*, *GATA3*, *PTEN*, *FGFR2*, *MKI67*, *SCUBE2*, *CCND1*, *EMSY*, *HBXAP*, *GAB2*, *PGR*, *BIRC2*, *HER3*, *MDM2*, *BRCA2*, *RB1*, *SPRY2*, *FOXA1*, *MTA1*, *RAD51*, *PTPN9*, *IGFR1*, *CDH3*, *CDH1*, *CD68*, *TP53*, *AURKB*, *ERBB2*/*HER2*, *GRB7*, *BECN1*, *BRCA1*, *MME1*, *TRIM25*, *BCAS3*, *BIRC5*, *BCL2*, *MYBL2*, *AIB1*, *AURKA*, and *MMP11*.

### Quantitative PCR (QPCR) analysis of *HER2* copy numbers

The QPCR analysis of *HER2* copy numbers was carried out with a Light Cycler (Roche Diagnostics, Indianapolis, IN) and TaqMan probe (Roche Diagnostics). We designed two regions of *HER2* [HER2 5´ region, 5´-GACAGCCGCAGTAGCTTCTTA-3´ and 5´-CAAAATGGAGCGCAGGTT-3´ (UPL#34); *HER2* 3´ region, 5´- GAGAACCCCGAGTACTTGACAC-3´ and 5´- CCAGTAATAGAGGTTGTCGAAGG-3´ (UPL#63)] to quantify the copy number of *HER2*. *MOCS2* at 5q11.2 and *SCN7A* at 2q24.3, where the normal copy was identified by a SNP array-based analysis, were used as reference genes.

### Quantitative reverse-transcription PCR (QRT-PCR) analysis of wild-type and variant *HER2* transcripts

First strand cDNA was synthesized as described previously and the quantification of *ACTB* mRNA was performed as a control to confirm the series of procedures [20]. QRT-PCR using StepOne Plus and MGB probes (Applied Biosystems, Foster City, CA) was used to quantify the wild-type and variant *HER2* mRNA, Δ*HER2*, containing an in-frame deletion. Primers used were; wild-type *HER2* 5´-TCCTGTGTGGACCTGGATGA-3´ and 5´-GACCAGCAGAATGCCAACCA-3´, probe 5´-AAGGGCTGCCCCGC-3´; Δ *HER*, 5´-CAACTGCACCCACTCCCC-3´, 5´-CTTGATGAGGATCCCAAAGACC-3´, probe 5´-CATCATCTCTGCGGTGGT-3´. The copy number for wild-type *HER2* or Δ*HER2* was calculated in absolute units by comparing the signal generated by the test samples to that generated by a set of external plasmid standards containing the sequence of wild-type *HER2* or Δ*HER2*[23,24]. The stock plasmid standard was created by ligating a PCR product containing the wild-type *HER2* or Δ*HER2* sequence into a plasmid vector system, pGEM-T Easy Vector System I (Promega, Madison, WI), according to the manufacturerΔs protocol. The amount of plasmid DNA was determined by spectrophotometric analyses of the insert-containing plasmid DNA at *A*_260_ (1 optical density = 50 Î¼g/ml plasmid DNA), and the copy number per milliliter was determined based on molecular weight. Dilutions of the plasmid ranged from 10 to 300,000 copies per reaction, and quantitative determination for clinical samples was carried out by reading from this standard curve.

### Analysis of *TP53* and *PIK3CA* mutations

To detect specific point mutations, genomic DNA from tumor samples was examined using PCR primers to cover exons 2–10 of *TP53*, and to cover exons 9 and 20 of *PIK3CA*. PCR products were directly sequenced with the BigDye Terminator v3.1 Cycle Sequencing Kit (Applied Biosystems).

### Fluorescence in situ hybridization (FISH)

Some tumors showed discordance between *HER2* genomic status determined with SNP arrays or *HER2* copy numbers examined by QPCR and IHC (HercepTest, DAKO) with or without FISH using paraffin specimens (PathVysion, Abbott, Japan). Some of these tumors were subsequently analyzed by FISH, using defrosted tumor specimens stamped on slide glasses. The chromosome 17 alpha satellite DNA was generated by PCR [25]. A BAC clone, RP11-62N23, was used for detection of the *HER2* region.

### Statistical analysis

Patients were classified into two groups based on presence or absence of alterations of 65 cancer-associated genes, and the two groups were further classified into four groups based on *HER2* genomic copy numbers, hormone receptor status (HR+/−), or the response to neoadjuvant chemotherapy (pCR vs. no pCR). Significance of differences in clinical and genetic characteristics between patientΔs groups was examined using the chi-square or FisherΔs exact test and StudentΔs t-test. RFS for each group of patients classified on the basis of clinical and genetic characteristics was estimated using the Kaplan-Meier method, and compared using the log-rank test. Time to failure was defined as the interval between diagnosis and the time of first recurrence or last follow-up. We also assessed the association between *HER2* genomic copy numbers and wild-type *HER2* or Δ16*HER2* mRNA levels by determining the Spearman rank correlation coefficient and associated *P*-value.

## Results

On the basis of routine methods, 77 tumors were classified as the HER2-/HR+ type, 28 as the HER2+/HR+ type, 18 as the HER2+/HR- type, and 20 as the HER2-/HR- type. The 46 HER2+/HR+ and HER2+/HR- tumors are the subjects of this paper. The numbers of four chromosome aberrations, including gain, loss, amplification and UPD, were examined in each tumor. Gain and amplification of oncogenes were individually described in Additional file 1: Table S1, however, they were combined and are referred to as gain in the following analyses. All the 46 tumors showed at least some chromosome aberrations.

### Discordance ratios between the results of the routine and definitive analyses on HER2 status

The routine method identified 46 tumors with the HER2+/HR+/− type. Of the 46 tumors, 39 were classified as HER2 IHC 3+ and 7 as HER2 IHC 2+ with positive routine FISH results. SNP array patterns for the *HER2* locus disclosed gain in 38, normal in 6, and UPD in two of the 46 tumors. Genomic *HER2* copy numbers were successfully examined in 45 of the 46 tumors by QPCR; 37 tumors showed *HER2* copy numbers with >2.0 indicating *HER2* gain, and 8 showed genome copy numbers between 0.5 and 2.0, indicating no gain of *HER2*. FISH analyses using defrosted tissue specimens were carried out in 11 tumors for the validation of the results of SNP array and QPCR analyses; SNP array patterns were normal or showed UPD in 7, and gain in four. The data obtained by the definitive FISH analysis were consistent with those obtained by SNP array and QPCR analyses (Additional file 1: Table S1). Accordingly, one tumor (No. 28), which was not examined by QPCR and identified to have *HER2* gain by SNP array, was included in the tumors with *HER2* copy numbers >2.0 for further analysis. Thus, 4 HER2 IHC 3+ (8.7%) and 4 HER2 IHC 2+ (8.7%) tumors of the 46 tumors showed *HER2* copy numbers ≤ 2.0 by QPCR, and nonamplified *HER2* by definitive FISH analysis.

### The relationship between *HER2* genomic copy numbers and clinical and genetic factors or levels of *HER2* mRNA

Patients having tumors with lower *HER2* copy numbers (≤ 2.0) showed more difficulty in achieving pCR than patients having tumors with higher *HER2* copy numbers (> 2.0) (*P*=0.019) (Table 1). Tumors with the lower *HER2* copy numbers had lower levels of wild-type *HER2* mRNA (<400) than tumors with the higher *HER2* copy numbers (*P*=0.035), and showed higher incidences of *PIK3CA* mutation (*P*=0.024), mutations and gain of *PIK3CA* (*P*=0.005), loss of *PTEN* (*P*=0.008), or a combined alteration of HER2 downstream genes, *PIK3CA*, *PTEN*, and *INPP4B* (P=0.009) than tumors with the higher *HER2* copy numbers with respective genetic alterations.

**Table 1:** **Differences in characteristics between two types of breast cancer classified by*HER2*copy numbers**

	***HER2*copy number ≤ 2.0 (n=8)**	***HER2*copy number > 2 (n=38)**	***P*-value**
Hormone receptor status (n=46)			
Positive	6	22	0.368
Negative	2	16	
Response to neoadjuvant chemotherapy (n=37)			
pCR (grade 3)	1	19	0.019
No pCR (grade 0–2)	6	11	
Relapse after surgery (n=42)			
Not occurred	3	27	0.018
Occurred	5	7	
Wild-type *HER2* mRNA (n=41)			
< 400	5	18	0.035
≥ 400	0	18	
Δ*HER2* mRNA (n=41)			
< 4.5	4	15	0.107
≥ 4.5	1	21	
Percentages of Δ*HER2* mRNA (n=41)			
< 2.4%	3	32	0.087
≥ 2.4%	2	4	
*PI3KCA* (exons 9 and 20) (n=46)			
Wild-type	5	35	0.024
Mutated	3	3	
*PI3KCA* (exons 9 and 20) (n=46)			
Wild-type + Normal + Loss + UPD	2	29	0.005
Mutated + Gain	6	9	
*PTEN* (n=46)			
Normal + Gain + UPD	5	36	0.008
Loss	3	2	
*INPP4B* (n=46)			
Normal + Gain + UPD	6	36	0.072
Loss	2	2	
*PI3KCA* , *PTEN*, *INPP4B* (n=46)			
No aberrations*	1	24	0.009
Aberrations**	7	14	
*DEK* (n=46)			
Normal	3	27	0.07
Gain	5	11	
*FGFR1* (n=46)			
Normal + Loss + UPD	4	27	0.248
Gain	4	11	
*CCND1* (n=46)			
Normal + Loss	4	21	0.786
Gain	4	17	
*FOXA1* (n=46)			
Normal + UPD	4	29	0.133
Gain	4	9	
*CDH3* (n=46)			
Normal + Loss + UPD	6	35	0.158
Gain	2	3	
*BIRC5* (n=46)			
Normal + Loss + UPD	6	23	0.441
Gain	2	15	
*MYBL2* (n=46)			
Normal + Loss	4	28	0.186
Gain	4	10	
*AIB1* (n=46)			
Normal + Loss	5	28	0.523
Gain	3	10	

*No aberrations, wild-type and a normal copy of *PIK3CA*, a normal copy, gain or UPD of *PTEN* or *INPP4B*; **Aberrations, mutated and/or gain of *PIK3CA*, and loss of *PTEN* or *INPP4B.*

*HER2* transcripts were successfully examined in 41 tumors, in all of which genomic copy numbers were also examined by QPCR. The Spearman rank correlation coefficient analysis showed that *HER2* genome copy numbers tended to be correlated with the levels of wild-type *HER*2 mRNA (rS=0.280, *P*=0.076) and Δ16*HER2* mRNA (rS=0.297, *P*=0.059), but not with the percentages of Δ16*HER2* mRNA (rs=−0.200, *P*=0.202). The expression levels of wild-type *HER2* mRNA were correlated with the expression levels of Δ16*HER2* mRNA (rS=0.901, *P*=1.05E-15).

### Genetic aberrations and *HER2* copy numbers and transcripts detected in HER2+/HR+ and HER2+/HR- tumors

Of 28 HR+ tumors, two were ER-negative/PgR-positive, 6 were ER-positive/PgR-negative, and 20 were ER-positive/PgR-positive. There were no significant differences in the frequency of three aberrations (gain, loss, and UPD) between 28 HER2+/HR+ tumors and 18 HER2+/HR- tumors. HR+/HER2+ tumors had lower levels of wild-type *HER2* mRNA (*P*=0.05) and Δ16*HER2* mRNA (*P*<0.001) and a lower incidence of *BIRC5* gain (*P*=0.018) than HER2+/HR- tumors (Table 2). In addition, HER2+/HR+ tumors tended to have higher incidences of *PTEN* loss (*P*=0.058) and *CDH3* gain (*P*=0.058) than HER2+/HR- tumors. If we excluded the 8 patients with nonamplified *HER2*, the same genes showed different incidences between the two types of patients (Table 2, numbers in parentheses).

**Table 2:** Differences in characteristics between two types of breast cancer classified by hormone receptor status

	**HER2+/HR+, n=28 (n=22)**	**HER2+/HR-, n=18 (n=16)**	***P*-value**
Response to neoadjuvant chemotherapy			
pCR (grade 3)	8 (8)	12 (11)	0.009 (0.034)
No pCR (grade 0–2)	14 (9)	3 (2)	
*HER2* copy numbers			
≤ 2.0	6	2	0.368
> 2.0	22	16	
Wild-type *HER2* mRNA			
< 400	16 (13)	7 (5)	0.05 (0.044)
≥ 400	7 (7)	11 (11)	
Δ*HER2* mRNA			
< 4.5	16 (13)	3 (2)	<0.001 (0.001)
≥ 4.5	7 (7)	15 (14)	
Percentages of Δ*HER2* mRNA			
< 2.4%	20 (18)	15 (14)	0.745 (0.813)
≥ 2.4%	3 (2)	3 (2)	
*PI3KCA* (exons 9 and 20)			
Wild-type	24 (19)	16 (14)	0.755 (0.418)
Mutated	4 (1)	2 (2)	
*PI3KCA* (exons 9 and 20)			
Wild-type + Normal + Loss + UPD	19 (18)	12 (12)	0.933 (0.611)
Mutated + Gain	9 (4)	6 (4)	
*PTEN*			
Normal + Gain + UPD	23 (20)	18 (16)	0.058 (0.215)
Loss	5 (2)	0 (0)	
*INPP4B*			
Normal + Gain + UPD	24 (20)	16 (14)	0.755 (0.735)
Loss	4 (2)	2 (2)	
*PI3KCA* , *PTEN*, *INPP4B*			
No aberrations*	15 (15)	10 (9)	0.895 (0.452)
Aberrations**	13 (7)	8 (7)	
*DEK*			
Normal	19 (17)	11 (11)	0.639 (0.556)
Gain	9 (5)	7 (5)	
*FGFR1*			
Normal + Loss + UPD	18 (15)	13 (12)	0.575 (0.871)
Gain	10 (7)	5 (4)	
*CCND1*			
Normal + Loss	16 (13)	9 (8)	0.635 (0.578)
Gain	12 (9)	9 (8)	
*FOXA1*			
Normal + UPD	20 (18)	13 (11)	0.953 (0.35)
Gain	8 (4)	5 (5)	
*CDH3*			
Normal + Loss + UPD	23 (19)	18 (16)	0.058 (0.124)
Gain	5 (3)	0 (0)	
*BIRC5*			
Normal + Loss + UPD	22 (17)	8 (6)	0.018 (0.013)
Gain	6 (5)	10 (10)	
*MYBL2*			
Normal + Loss	18 (15)	14 (13)	0.332 (0.366)
Gain	10 (7)	4 (3)	
*AIB1*			
Normal + Loss	19 (15)	14 (13)	0.466 (0.366)
Gain	9 (7)	4 (3)	

Numbers in parentheses indicate patientsΔ numbers excluding 6 HER2+/HR+ and 2 HER2+/HR- patients whose tumors had *HER2* copy numbers ≤ 2.0, and *P*-values calculated from the numbers in the parentheses.

*No aberrations, wild-type and a normal copy of *PIK3CA*, a normal copy, gain or UPD of *PTEN* or *INPP4B*; **Aberrations, mutated and/or gain of *PIK3CA*, and loss of *PTEN* or *INPP4B.*

### Response to neoadjuvant chemotherapy and RFS between two groups of patients classified by clinical and genetic characteristics in tumors

The response to neoadjuvant chemotherapy and RFS were evaluated in 37 and 42, respectively, of 46 patients (Table 3). Clinical factors, including age and clinical T and N stages, did not affect the response to neoadjuvant chemotherapy or RFS (data not shown). HR+ tumors showed more difficulty in achieving pCR than HR- tumors (*P*=0.009). Tumors with higher copy numbers of *HER2* and higher levels of Δ16*HER2* mRNA entered pCR more frequently than their counterparts (*P*=0.019 and *P*=0.020). Tumors with gain of *FGFR1* or *MYBL2* had more difficulty in achieving pCR than the tumors without (*P*=0.035 and *P*=0.028). Tumors with loss of *PTEN* had more difficulty entering pCR than the tumors without (*P*=0.009). Tumors with a combination of mutations and gain of *PIK3CA*, or combined aberrations of the PI3K pathway genes, *PIK3CA*, *PTEN* and *INPP4B* tended to have more difficulty entering pCR than tumors without (*P*=0.08 and *P*=0.086).

**Table 3:** RFS and pCR rates for 42 patients classified by clinical and genetic characteristics

	**Response to neoadjuvant chemotherapy**			**Relapse-free survival**			
	**pCR**	**No pCR**	***P*-value**	**No. of Patients (No. of events)**	**3-year estimates**	**95% CI**	***P*-value**
All patients	20	17		42 (12)	0.72	0.55-0.89	
Response to neoadjuvant chemotherapy							
pCR (grade 3)				20 (4)	0.73	0.52-0.93	0.764
No pCR (grade 0~2)				17 (5)	0.86	0.67-1.04	
Hormone receptors							
Positive	8	14	0.009	25 (7)	0.76	0.58-0.95	0.914
Negative	12	3		17 (5)	0.69	0.42-0.95	
*HER2* copy numbers							
≤ 2.0	1	6	0.019	8 (5)	0.57	0.20-0.94	0.095
> 2.0	19	11		34 (7)	0.78	0.62-0.94	
Wild-type *HER2* mRNA							
< 400	8	9	0.208	20 (8)	0.61	0.40-0.83	0.022
≥ 400	11	5		17 (2)	0.92	0.76-1.07	
∆*16HER2* mRNA							
< 4.5	5	8	0.020	17 (5)	0.72	0.50-0.93	0.314
≥ 4.5	15	4		20 (5)	0.75	0.53-0.96	
Percentages of ∆*16HER2* mRNA							
< 2.4%	17	11	0.581	31 (6)	0.79	0.63-0.94	0.039
≥ 2.4%	5	1		6 (4)	0.50	0.10-0.90	
*PIK3CA*							
Wild-type	18	14	0.498	36 (9)	0.79	0.64-0.94	0.171
Mutated	2	3		6 (3)	0.42	−0.02-0.85	
*PIK3CA*							
Wild-type + Normal + Loss + UPD	16	9	0.08	28 (6)	0.85	0.70-1.01	0.041
Mutated + Gain	4	8		14 (6)	0.56	0.29-0.83	
*PTEN*							
Normal + Gain + UPD	20	12	0.009	37 (11)	0.73	0.56-0.89	0.705
Loss	0	5		5 (1)	0.80	0.45-1.15	
*INPP4B*							
Normal + Gain + UPD	17	16	0.373	36 (8)	0.79	0.64-0.94	0.095
Loss	3	1		6 (4)	0.50	0.10-0.90	
*PI3KCA , PTEN, INPP4B*							
No aberrations*	14	6	0.086	22 (4)	0.81	0.60-1.01	0.195
Aberrations**	6	11		20 (8)	0.67	0.45-0.89	
*DEK*							
Normal	13	14	0.236	28 (5)	0.88	0.75-1.01	0.006
Gain	7	3		14 (7)	0.44	0.14-0.75	
*FGFR1*							
Normal + Loss + UPD	17	9	0.035	29 (9)	0.75	0.48-0.86	0.641
Gain	3	8		13 (3)	0.92	0.76-1.07	
*CCND1*							
Normal + Loss	11	12	0.33	25 (4)	0.84	0.68-1.01	0.043
Gain	9	5		17 (8)	0.58	0.33-0.84	
*FOXA1*							
Normal + UPD	15	13	0.917	30 (6)	0.80	0.64-0.96	0.012
Gain	5	4		12 (6)	0.60	0.29-0.91	
*CDH3*							
Normal + Loss + UPD	19	14	0.217	37 (8)	0.79	0.64-0.94	0.009
Gain	1	3		5 (4)	0.40	−0.03-0.83	
*BIRC5*							
Normal + Loss + UPD	14	13	0.659	27 (5)	0.82	0.66-0.98	0.005
Gain	6	4		15 (7)	0.56	0.25-0.88	
*MYBL2*							
Normal + Loss	18	10	0.028	30 (7)	0.80	0.64-0.96	0.053
Gain	2	7		12 (5)	0.58	0.24-0.91	
*AIB1*							
Normal + Loss	18	11	0.063	31 (7)	0.81	0.65-0.96	0.017
Gain	2	6		11 (5)	0.52	0.15-0.89	

CI, confidence interval; *No aberrations, wild-type and a normal copy of *PIK3CA*, a normal copy, gain or UPD of *PTEN* or *INPP4B*; **Aberrations, mutated and/or gain of *PIK3CA*, and loss of *PTEN* or *INPP4B.*

**Figure 1:**
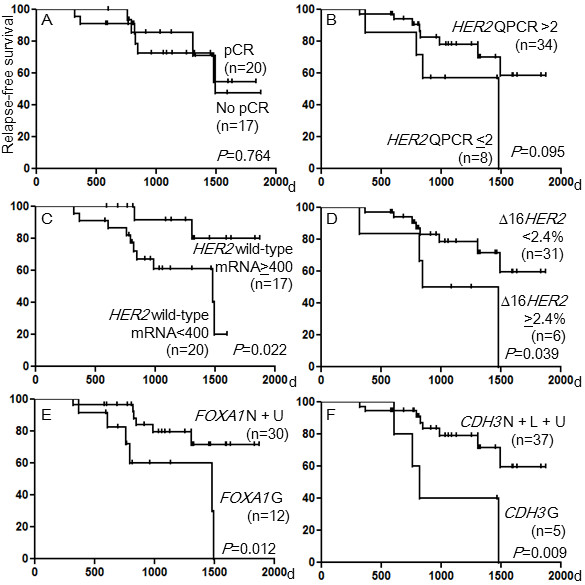
**Relapse-free survival curves for patients classified by clinical and genetic characteristics.** (**A**) Patients who achieved pCR and those who did not, (**B**) patients with *HER2* copy numbers ≤ 2.0 and those with *HER2* copy numbers >2.0 in the tumors, (**C**) patients with wild-type *HER2* mRNA < 400 and those with wild-type *HER2* mRNA ≥ 400 in the tumors, (**D**) patients with Δ16*HER2* mRNA < 2.4% and those with Δ16*HER2* mRNA ≥ 2.4% in the tumors, (**E**) patients with gain of *FOXA1* and those with a normal copy or UPD of *FOXA1* in the tumors, (**F**) patients with gain of *CDH3* and those with a normal copy, loss, or UPD of *CDH3* in the tumors.

**Figure 2:**
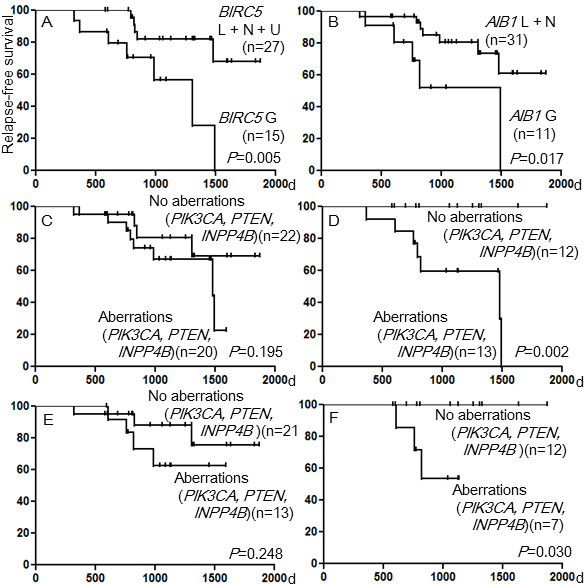
**Relapse-free survival curves for patients classified by genetic characteristics.** (**A**) Patients with gain of ***BIRC5*** and those with a normal copy, loss, or UPD of *BIRC5* in the tumors, (**B**) patients with gain of *AIB1* and those with a normal copy or loss of *AIB1* in the tumors, (**C**) 20 patients with HR+ or HR- tumors with combined aberrations of *PIK3CA*, *PTEN*, and *INPP4*B, and 22 patients with HR+ or HR- tumors with no aberrations of *PIK3CA*, *PTEN*, and *INPP4B*, (**D**) 13 patients with HR+ tumors with combined aberrations of *PIK3CA*, *PTEN*, and *INPP4B*, and 12 patients with HR+ tumors with no aberrations of *PIK3CA*, *PTEN*, and *INPP4B*, (**E**) 13 patients with HR+ or HR- tumors with combined aberrations of *PIK3CA*, *PTEN*, and *INPP4B*, and 21 patients with HR+ or HR- tumors with no aberrations of *PIK3CA*, *PTEN*, and *INPP4B*, and (**F**) 7 patients with HR+ tumors with combined aberrations of *PIK3CA*, *PTEN*, and *INPP4B*, and 12 patients with HR+ tumors with no aberrations of *PIK3CA*, *PTEN*, and *INPP4B*.

The median follow-up time of the 42 patients was 41.2 months ranging from 19.3 to 85.3 months. There was no difference in RFS between patients who achieved pCR and those who did not (*P*=0.764) (Figure 1A) (Table 3). Patients with lower *HER2* genome copy numbers tended to have worse RFS rates than those with higher *HER2* genome copy numbers (*P*=0.095) (Figure 1B). In regard to *HER2* transcripts, patients with lower levels of the wild-type *HER2* mRNA (< 400) in tumors showed worse RFS rates than those with higher levels of the wild-type mRNA (≥ 400) (*P*=0.022) (Figure 1C), and patients with higher percentages of the Δ16*HER2* transcript (≥ 2.4%) in tumors showed worse RFS rates than those with lower percentages (< 2.4%) (*P*=0.039) (Figure 1D). Patients with a combination of mutations and gain of *PIK3CA* (*P*=0.041), gain of *DEK* (*P*=0.006), *CCND1* (*P*=0.043)), *FOXA1* (*P*=0.012) (Figure 1E), *CDH3* (*P*=0.009) (Figure 1F), *BIRC5* (*P*=0.005) (Figure 2A), or *AIB1* (*P*=0.017) (Figure 2B) in tumors had worse RFS rates than patients without.

Next, we evaluated the pCR and RFS rates in 30 and 34 patients, respectively, excluding 7 and 8 patients with *HER2* copy numbers ≤ 2.0 from the 37 and 42 patients, respectively, because data on patients only with a *HER2*-amplified tumor may be important to show outcome of the therapy given to a specified group of patients (Additional file 2 Table S2). The positive HR status (*P*=0.034), loss of *PTEN* (*P*=0.054), and gain of *FGFR1* (*P*=0.077), *MYBL2* (*P*=0.088) or *AIB1* (*P*=0.088) indicated or tended to indicate more difficulty in achieving pCR than the respective counterparts. In contrast, predictive significance of Δ16*HER2* < 4.5 for difficulty in achieving pCR disappeared (*P*=0.132), and that of *DEK* gain for more likely to achieve pCR newly appeared (*P*=0.037). In regard to RFS, patients with wild-type *HER2* mRNA < 400 (*P*=0.066), mutation and gain of *PIK3CA* (*P*=0.06), gain of *CDH3* (*P*<0.001), *BIRC5* (*P*=0.007), *MYBL2* (*P*=0.013), or *AIB1* (*P*=0.013) had or tended to have worse RFS rates than those without. In contrast, the prognostic significance of Δ16*HER2* ≥ 2.4% (*P*=0.1) and gain of *DEK* (*P*=0.108), *CCND1* (*P*=0.247), or *FOXA1* (*P*=0.217) found in the 42 patients disappeared in the 34 patients. Thus, the studies including or excluding the 8 patients showed or suggested prognostic significance of certain genetic alterations, especially those involved in the PI3K and ER pathways.

### Genetic characteristics that show significant differences in RFS between HR+ tumors and HR- tumors

Patients having a HR+ tumor with mutations of *PIK3CA* (*P*=0.006), a combination of mutations and gain of *PIK3CA* (*P*=0.001), a combined aberration of *PIK3CA*, *PTEN* and *INPP4B* (*P*=0.002), and gain of *FOXA1* (*P*=0.002), *CDH3* (*P*=0.007), *BIRC5* (*P*=0.016), *MYBL2* (*P*=0.015), and *AIB1* (*P*=0.006) had worse RFS rates than patients having a HR+ tumor without (Table 4). However, no such significance of the genetic aberrations was found in patients with a HR- tumor. It is noteworthy that while there was no significant difference in a RFS rate between 20 patients having a HR+ or HR- tumor with aberrations of the PI3K pathway genes and 22 patients having a HR+ or HR- tumor with no such aberrations (Table 3 and Figure 2C, *P*=0.195), 13 patients having a HR+ tumor with the same aberrations had a worse RFS rate than 12 patients having a HR+ tumor with no such aberrations (Table 4, Figure 2D, *P*=0.002). When we excluded the 8 patients with *HER2* copy numbers ≤ 2.0 in tumors, the prognostic significance of the genetic alterations was also demonstrated in 19 patients with a HR+ tumor (Additional file 3: Table S3, Figure 2F, *P*=0.03), but not in the 34 patients with a HR+ or HR- tumor (Additional file 2: Table S2, Figure 2E, *P*=0.248).

**Table 4:** RFS in patients with a HR-positive or HR–negative tumor classified by clinical and genetic characteristics

	**Hormone receptor-positive tumors (n=25)**				**Hormone receptor-negative tumors (n=17)**			
	**No. of Patients (No. of events)**	**3-year estimates**	**95% CI**	***P*-value**	**No. of Patients (No. of events)**	**3-year estimates**	**95% CI**	***P*-value**
All patients	25 (7)	0.76	0.58-0.95		17 (5)	0.69	0.42-0.95	
Response to neoadjuvant CT								
pCR (grade 3)	8 (1)	0.81	0.58-1.05	0.485	12 (3)	1.0	-	0.906
No pCR (grade 0~2)	14(4)	0.77	0.49-1.05		3 (1)	0.69	0.39-0.99	
*HER2* copy numbers								
≤ 2.0	6 (4)	0.60	0.17-1.03	0.12	2 (1)	0.50	−0.19-1.19	NA
> 2.0	19 (3)	0.81	0.62-1.01		15 (4)	0.74	0.47-1.00	
Wild-type *HER2* mRNA								
< 400	14 (5)	0.67	0.43-0.91	0.054	6 (3)	0.33	−0.17-0.83	0.196
≥ 400	6 (0)	1.0	-		11 (2)	0.86	0.60-1.12	
∆*16HER2* mRNA								
< 4.5	14 (4)	0.74	0.53-0.96	0.283	3 (1)	0	0	0.832
≥ 4.5	6 (1)	0.80	0.45-1.15		14 (4)	0.72	0.45-1.00	
Percentages of ∆*16HER2* mRNA								
< 2.4%	17 (3)	0.78	0.59-0.97	0.143	14 (3)	0.77	0.49-1.00	0.073
≥ 2.4%	3 (2)	0.67	0.13-1.20		3 (2)	0.33	−0.20-0.87	
*PIK3CA* (exons 9 and 20)								
Wild-type	21 (4)	0.89	0.75-1.03	0.006	15 (5)	0.65	0.37-0.94	NA
Mutated	4 (3)	0.25	−0.17-0.67		2 (0)	1.0	-	
*PIK3CA* (exons 9 and 20)								
Wild-type + Norma l+ Loss +UPD	16 (1)	1.0	-	0.001	12 (5)	0.57	0.25-0.89	0.2
Mutated + Gain	9 (6)	0.40	0.06-0.74		5 (0)	1.0	-	
*PTEN*								
Normal + Gain + UPD	20 (6)	0.76	0.55-0.97	0.814	17 (5)	0.69	0.42-0.95	NA
Loss	5 (1)	0.80	0.45-1.15		0	-	-	
*INPP4B*								
Normal + Gain + UPD	21 (4)	0.83	0.65-1.01	0.133	15 (4)	0.75	0.49-1.00	NA
Loss	4 (3)	0.50	0.01-0.99		2 (1)	0.50	−0.19-1.19	
*PI3KCA, PTEN, INPP4B*								
No aberrations*	12 (0)	1.0	-	0.002	10 (4)	0.59	0.23-0.95	0.312
Aberrations**	13 (7)	0.60	0.32-0.87		7 (1)	0.75	0.33-1.17	
*DEK*								
Normal	17 (3)	0.86	0.68-1.04	0.067	11 (2)	0.91	0.74-1.08	0.106
Gain	8 (4)	0.51	0.11-0.91		6 (3)	0.30	−0.17-0.77	
*FGFR1*								
Normal + Loss + UPD	17 (5)	0.72	0.49-0.96	0.94	12 (4)	0.59	0.28-0.91	0.69
Gain	8 (2)	0.86	0.60-1.12		5 (1)	1.0	-	
*CCND1*								
Normal + Loss	16 (2)	0.91	0.74-1.08	0.075	9 (2)	0.71	0.36-1.06	0.433
Gain	9 (5)	0.51	0.16-0.85		8 (3)	0.67	0.29-1.04	
*FOXA1*								
Normal + UPD	17 (1)	0.92	0.76-1.07	0.002	13 (5)	0.66	0.39-0.93	0.504
Gain	8 (6)	0.45	0.08-0.82		4 (0)	1.0	-	
*CDH3*								
Normal + Loss + UPD	20 (3)	0.88	0.73-1.04	0.007	17 (5)	0.69	0.42-0.95	NA
Gain	5 (4)	0.40	−0.03-0.83		0	-	-	
*BIRC5*								
Normal + Loss + UPD	19 (3)	0.86	0.68-1.04	0.016	8 (2)	0.75	0.45-1.05	0.235
Gain	6 (4)	0.44	0.01-0.88		9 (3)	0.59	0.10-1.09	
*MYBL2*								
Normal + Loss	16 (2)	0.92	0.78-1.07	0.015	14 (5)	0.66	0.39-0.94	0.539
Gain	9 (5)	0.48	0.11-0.84		3 (0)	1.0	-	
*AIB1*								
Normal + Loss	17 (2)	0.93	0.79-1.06	0.006	14 (5)	0.66	0.39-0.94	0.539
Gain	8 (5)	0.39	−0.01-0.78		3 (0)	1.0	-	

CI, confidence interval; CT, chemotherapy; NA, not applicable; *No aberrations, wild-type and a normal copy of *PIK3CA*, a normal copy, gain, or UPD of *PTEN* or *INPP4B*; **Aberrations, mutated and/or gain of *PIK3CA*, and loss of *PTEN* or *INPP4B.*

### Clinical and genetic characteristics that predict pCR or RFS, or both

Patients with higher *HER2* copy numbers, a combination of mutations and gain of *PIK3CA*, and gain of *MYBL2* and *AIB1* had or tended to have worse pCR and RFS rates than the respective counterparts (Table 3). In contrast, patients with positive HR status, lower levels of Δ16*HER2* transcript, loss of *PTEN*, and gain of *FGFR1* had more difficulty in achieving pCR, but not worse RFS rates than the respective counterparts, whereas patients with lower levels of wild-type *HER2* mRNA, higher percentages of Δ16*HER2* mRNA, and gain of *DEK*, *CCND1*, *FOXA1*, *CDH3*, or *BIRC5* had worse RFS rates, but not more difficulty in achieving pCR than the respective counterparts. Gain of *MYC*, *EMSY*, and *AURKA*, *TP53* mutations, and other genetic aberrations did not affect pCR or RFS (data not shown). Thus, some genetic aberrations indicated either the response to the chemotherapy or the RFS, and others indicated both. These findings also indicated that genetic aberrations that correlate with difficulty in achieving pCR and those that correlate with worse RFS rates do not always overlap.

### Correlation of immunohistochemical findings of *CDH3* with hormone receptor status, genetic alterations, and RFS

All 5 tumors with gain of *CDH3* belonged to the HR+ type, and patients having a tumor with gain of *CDH3* had worse RFS rates than patients without. To validate the findings shown by the SNP array, we performed immunohistochemical staining of P-cadherin encoded by *CDH3*. While 5 tumors with *CDH3* gain showed 1+, 2+, or 3+ P-cadherin expression, 8 of 33 tumors with *CDH3* loss or a normal *CDH3* genetic status showed negative P-cadherin expression. While all 18 HR- tumors showed 1+, 2+, or 3+ P-cadherin expression, 8 of 22 HR+ tumors showed negative P-cadherin expression (*P*<0.01). Patients with P-cadherin 3+ expression/HR+ tumors had worse RFS rates than patients with *P*-cadherin negative or 1+ expression/HR+ tumors (*P*=0.032). Thus, while all HR- tumors showed P-cadherin expression, some HR+ tumors were negative for P-cadherin expression, indicating that the staining patterns and their prognostic implications differed between the two types of tumors.

## Discussion

In the present SNP array-based study of 46 HER2+ breast cancers, we evaluated clinical and genetic factors that indicate pCR and RFS in patients who were treated with trastuzumab-containing neoadjuvant chemotherapy. SNP array analysis has a merit to detect whole genomic aberrations at once, and therefore could find a predictive or prognostic impact of combined genomic aberrations. Such an attempt seems not to have been made before. The 46 tumors were selected based on a routine HER2 study using IHC with or without FISH in deparaffinized tissue samples. We found discordance between results of the routine analysis and the present SNP and QPCR analysis with FISH in defrosted tissue samples in 17.4% (8/46) of tumors; 4 IHC 3+ (8.7%) and 4 IHC 2+ (8.7%) tumors. Previous studies reported the IHC3+, FISH negative type in 0.9%-18.5% of tumors examined [9], and the percentage of 8.7% shown in the present study may be comparable to or a little higher than the previous results. Another discordance of 4 patients with the IHC 2+ and nonamplified *HER2* type may be caused by the difficulty of FISH analysis using needle biopsied tissues embedded in paraffin. It is difficult to compare this percentage of 8.7% with those of other series, because there have been no comparable studies reported.

Overexpression of protein occurs not only by mRNA overexpression, but also by post-transcriptional, translational and protein degradation regulation [26]. HER2 is efficiently ubiquitinated and downregulated by the chaperone binding ubiquitin ligase CHIP/STUB1 [27]. Recently, Jan et al. studied the expression and correlations among TID1, CHIP, and HER2 in a total of 183 breast cancer histology sections using IHC and immunoblotting assay, and found that the immunohistochemical expression of TID1 and CHIP were positively correlated with each other but were both inversely correlated to that of HER2 [28]. These findings suggest that down-regulation of CHIP could cause overexpression of HER2 by preventing its degradation. In a study of metastatic breast cancer with the HER2 IHC2+/3+ and nonamplified *HER2* type, patients treated with trastuzumab plus chemotherapy had better progression-free survival than those treated with chemotherapy alone (*P*=0.03), suggesting the presence of the true single-gene overexpressers in the study population [11]. Thus, some *HER2*-single-gene overexpressers were not the results of technical shortcomings of HER2 IHC, but could be the results of disruption of HER2 protein degradation occurring in the tumor cells. Overexpression of MYCN caused by the disruption of the protein degradation but not by the amplification indicated poor outcome in patients with neuroblastoma [29].

The present study indicated that the patients with nonamplified *HER2* showed more difficulty in achieving pCR and more frequent relapse than those with *HER2* amplification. Unexpectedly, we found that tumors with the lower *HER2* copy numbers had more frequent alterations in the PI3K pathway genes than those with the higher copy numbers (Table 1). Alterations of the PI3K pathway genes were reported to be associated with poor response to HER2-targeted therapy in patients with HER2+ tumors [30], and this finding concurs with the present finding indicating the poor outcome of patients having a tumor with HER2 IHC 2+/3+ and nonamplified *HER2*. These findings support the statement that FISH analysis should be the primary HER2 testing for patients who are candidates for HER2-targeted therapies [9].

In regard to the relationship between clinical and genetic factors and RFS, patients with various genetic changes in tumors identified by SNP array had or tended to have worse RFS rates than those without (Table 3). Previous studies reported that overexpression of these genes identified by various methods was associated with a poor outcome in patients with breast cancer [31-37]. Thus, the present study indicated that the results of the SNP array analysis detecting gain of specific genes and those of other analyses detecting overexpression of the gene products were mostly consistent.

When the prognostic implications of the genetic aberrations were separately examined in HR+ and HR- tumors, a combination of mutations and gain of *PIK3CA*, a combined aberration of the PI3K pathway genes, and gain of *FOXA1*, *CDH3*, *BIRC5*, *MYBL2*, and *AIB1* indicated worse RFS rates only in patients with a HR+ tumor (Table 4). It is noteworthy that the incidences of the most genetic changes did not differ between the two types of tumors (Table 2). We confirmed the result of a study reporting that increased PI3K pathway activity was associated with poor outcome for patients treated with HER2-targeting therapy [30], and newly found that the prognostic significance of the alterations in the PI3K and ER pathway genes applied only to the patients with a HR+ tumor (Table 4). FOXA1, a forkhead family transcription factor, is essential for optimum expression of ER and estrogen responsive genes. Although one study reported that FOXA1 expression was correlated with the luminal A subtype and a favorable outcome [38], others depicted a complicated picture of FOXA1 as a participant in multiple signaling pathways in breast cancer, which are both oncogenic and tumor suppressive [33]. Actually, a recent study reported co-expression of ER and FOXA1 in metastatic breast cancer samples, indicating oncogenic activities of *FOXA1*[39]. AIB1 is a nuclear receptor coactivator that interacts with ERs in a ligand-dependent fashion and enhances estrogen-dependent transcription. Harigopal et al. reported AIB1 activation to be mediated by crosstalk with other signaling kinases such as growth factor receptors including MAPK, which can be activated by HER2 in a ligand-independent fashion [40]. AIB1 is considered as a key factor in the regulation of tumor growth and carcinogenesis. In addition, patients with AIB1-positive tumors identified by IHC showed worse RFS rates than those with AIB1-negative tumors [40]. These findings indicate that crosstalk between the PI3K and ER pathways is causally involved in resistance to the chemotherapy with trastuzumab, and thus, that hormone receptor status impacted the prognostic significance of genomic alterations in HER2+ tumors.

The present study also showed that gain of *DEK* and *BIRC5* genes, which seems not to be involved in the PI3K or ER pathway, had clear prognostic significance in patients with breast cancer (Table 3), suggesting presence of other pathways exhibiting crosstalk with the PI3K or ER pathway. Lu et al. reported that HER2-mediated up-regulation of survivin expression contributed to Taxol resistance through a survivin-mediated faster exit from mitosis [41]. Xia et al. showed that acquired resistance to lapatinib in the BT474 cell line was associated with a switch in the regulation of survivin from HER2 to ER [42]. Thus, various roles of survivin in chemotherapy resistance may contribute to the prognostic significance of *BIRC5* gain in the present series of patients with HR+ and HR- breast cancers.

A splice variant of the human *HER2* transcript lacking exon 16 (Δ16*HER2*) has been detected in human breast cancers [12]. Mitra and colleagues showed that ectopic expression of Δ16*HER2*, but not wild-type *HER2* mRNA, promoted receptor dimerization, cell invasion, and trastuzumab resistance in the NIH3T3 and MCF7 cell lines [13]. Recently, a mouse line that transgenically expresses the Δ16*HER2* transcript has been generated and all the transgenic females developed multifocal mammary tumors with a rapid onset [14]. This oncogenic isoform has been associated with trastuzumab resistance in various in vitro and transgenic mouse studies [12,14]. The present study showed that levels of the wild-type *HER2* transcript and those of the Δ16*HER2* transcript were correlated, and that there was no difference in the RFS rate between patients with higher levels of Δ16*HER2* mRNA and those without. In addition, we also found that patients with higher percentages of the Δ16*HER2* mRNA (≥ 2.4%) and lower levels of the wild-type *HER2* mRNA (< 400) were likely to have recurrence. The decreased RFS rates for patients with lower levels of wild-type *HER2* mRNA may be explained by the decreased susceptibility to trastuzumab in patients with such tumors. The patients with the higher percentage of Δ16*HER2* mRNA also had the lower level of wild-type *HER2* mRNA, and this lower level might have resulted in a lower denominator and hence the higher percentages of Δ16*HER2* mRNA. Thus, the poor prognosis of patients with the higher percentage of Δ16*HER2* mRNA may be related to the lower levels of the wild-type *HER2* mRNA, or to presently unknown mechanisms.

The present study showed no difference in RFS between patients who attained pCR and those who did not. Previous studies indicated better overall survivals for patients with pCR than those with no pCR [2,3]. von Minckwits and colleagues studied pCR and its prognostic impact on survival in intrinsic breast cancer subtypes and concluded that pCR is a suitable surrogate end point for patients with HER2+ (nonluminal) but not luminal B/HER2+ breast cancer [43]. More recently, results of a meta-analysis showed that patients with a HR-/HER2+ or HR+/HER2+ tumor who achieved pCR had greater event-free survival than those with the respective subtype of tumor who did not [44]. The contradictory finding of the present study might be caused by the small sample size and inclusion of HR+/HER2+ and HR-/HER2+ breast cancer samples.

Several clinical trials have been carried out to improve the outcome of patients with HER2+ tumors. Two trials reported that the combination of an aromatase inhibitor with trastuzumab or lapatinib improved outcome for patients with HER2+/HR+ metastatic breast cancer compared with an aromatase inhibitor alone [45,46]. Both trials showed better progression-free survival for the patients treated with the combination. These studies were undertaken because the crosstalk between the PI3K and ER pathways could cause resistance to trastuzumab and anti-hormone agents. In addition, one phase 2 and another phase 3 clinical trials were carried out using a combination of lapatinib and trastuzumab with or without cytotoxic chemotherapy for patients with a HER2+/HR+ or HER2+/HR- early stage tumor, and both studies showed improved pCR rates [47,48]. These studies were undertaken because primary and acquired resistance to both agents could be overcome, their partly non-overlapping mechanisms of action, and the well-characterized synergistic interaction between them could be expected in patients with HER2+ breast cancer. The present study showed that the PI3K and ER pathway genes were specifically altered in HER2+/HR+ tumors of patients who subsequently relapsed after receiving neoadjuvant chemotherapy with trastuzumab. Lapatinib targets the intracellular ATP domain of HER2, preventing self-phosphorylation and subsequent activation of the PI3K and MAPK signal pathways [5]. It may be reasonable to add lapatinib to the present trastuzumab-containing chemotherapy for patients with a HER2+/HR+ tumor to overcome the resistance due to the altered PI3K pathway genes and to obtain a better outcome.

## Conclusion

The present study showed that 17.4% of tumors with positive HER2 testing had nonamplified *HER2*, and patients with this type of tumor showed difficulty in achieving pCR and frequent relapses, supporting FISH analysis as a primary HER2 testing. The study also disclosed that alterations of the PI3K and ER pathway genes indicated worse RFS rates in patients with a HR+ but not with a HR- tumor who were treated with neoadjuvant chemotherapy with trastuzumab. It may be reasonable to add lapatinib to the present trastuzumab-containing chemotherapy for patients with a HER2+/HR+ tumor to overcome the resistance due to the altered PI3K pathway genes and to obtain a better outcome.

## Abbreviations

ER: Estrogen receptor; FISH: Fluorescence in situ hybridization; H & E: Haematoxylin and eosin; HER2: Human epidermal growth factor receptor 2; HR: Hormone receptor; IHC: Immunohistochemistry; pCR: Pathological complete response; RFS: Relapse-free survival; PI3K: Phosphatidylinositol 3-kinase; SNP: Single nucleotide polymorphisms

## Competing interests

The authors declare that they have no competing interests.

## AuthorsΔ contributions

MT, TH, KT, MH, and JW participated in data collection, interpretation and molecular analysis. FK and MK carried out molecular cytogenetic and pathological studies. ASO and OS were responsible for SNP array analysis. MT, HT, KI, MM, and YK contributed to concept design and drafted the manuscript. All authors have read and approved the manuscript.

## Pre-publication history

The pre-publication history for this paper can be accessed here:

http://www.biomedcentral.com/1471-2407/13/241/prepub

## Supplementary Material

Additional file 1: Table S1 Clinical and genetic characteristics of 28 patients with a breast cancer of HER2+/HR+ type and 18 with a breast cancer of HER2+/HR- type. Click here for file

Additional file 2: Table S2 RFS and pCR rates for 34 patients with a HR+ or HR- tumor classified by clinical and genetic characteristics; 8 patients with HER2 copy numbers ≤ 2.0 are excluded. Click here for file

Additional file 3: Table S3 RFS in patients with a HR+ or HR- tumor classified by clinical and genetic characteristics; 8 patients with *HER2* copy numbers ≤ 2.0 are excluded. Click here for file
