# Supplementary material for: Alterations of the genes involved in the PI3K and estrogen-receptor pathways influence outcome in human epidermal growth factor receptor 2-positive and hormone receptor-positive breast cancer patients treated with trastuzumab-containing neoadjuvant chemotherapy
Source: BMC Cancer. 2013 May 16;13:241. doi: 10.1186/1471-2407-13-241 (PMC3663661; doi:10.1186/1471-2407-13-241)
Supplement: Additional file 1: Table S1 — Clinical and genetic characteristics of 28 patients with a breast cancer of HER2+/HR+ type and 18 with a breast cancer of HER2+/HR- type. [file 1471-2407-13-241-S1.docx]

Table S1. Clinical and genetic characteristics of 28 patients with a breast cancer of HER2+/HR+ type and 18 with a breast cancer of HER2+/HR- type

|  |  |  | *HER2* |  |  |  |  |  |  |  |  |  |  |  |  |  |  |  |  |  |
| --- | --- | --- | --- | --- | --- | --- | --- | --- | --- | --- | --- | --- | --- | --- | --- | --- | --- | --- | --- | --- |
| Patients number | Res. | Rel. | Copy N. | FISH  HER2/cen | Wild-type mRNA | Δ16 mRNA | %Δ16 | *HER2* | *PIK3CA* | *PIK3CA mutation* | *PTEN* | *INPP4* | *DEK* | *CCND1* | *FOXA1* | *CDH3* | *BIRC5* | *MYBL2* | *AIB1* | *TP53* |
| HER2+/HR+ type (n=28) | | | |  |  |  |  |  |  |  |  |  |  |  |  |  |  |  |  |  |
| 1 | N.E | + | 0.5 | 2/4 | N. D. | N. D. | N. D. | U | G | p.E545K | N | L | G | G | G | L | G | G | G |  |
| 2 | 1b | + | 0.7 | 2/2 | 24 | 1.4 | 6.10% | N | G |  | N | N | N | N | G | G | N | N | N | ex8/ms |
| 3 | 1a | - | 1.4 | 2/2 | N. D. | N. D. | N. D. | U | G |  | L | G | N | N | U | G | U | G | N |  |
| 4 | 2 | - | 1.6 | 3/3 | 40 | 0.8 | 2.10% | N | N | p.H1047D | L | N | N | N | N | N | N | N | N |  |
| 5 | 1b | + | 1.7 | 4/4 | N. D. | N. D. | N. D. | N | N | p.G1050S | L | N | G | A | G | N | N | N | N |  |
| 6 | 1a | + | 2 | 3/3 | 37 | 0.2 | 0.60% | N | N |  | N | L | G | G | G | N | G | G | G |  |
| 7 | N.E | - | 2.5 | 6/3 | 946 | 11.8 | 1.20% | L | N |  | N | N | N | A | N | L | L | N | N |  |
| 8 | N.E | - | 2.8 |  | 1347 | 4.2 | 0.30% | A | N |  | N | N | N | N | A | N | A | N | N |  |
| 9 | 3 | - | 3 |  | 9 | 0.2 | 2.00% | A | N |  | N | N | N | N | N | N | N | N | N |  |
| 10 | 2 | - | 3.1 |  | 403 | 1.4 | 0.40% | A | N |  | N | N | N | N | N | N | N | N | N |  |
| 11 | 1b | - | 3.8 |  | 72 | 0.6 | 0.80% | A | N |  | L | N | N | A | N | L | GU | G | G |  |
| 12 | 2 | - | 3.8 |  | 714 | 5.4 | 0.80% | A | N |  | N | N | N | N | N | L | L | N | N |  |
| 13 | 2 | - | 4.2 |  | 191 | 2.5 | 1.30% | A | N |  | N | N | N | N | N | N | N | N | N |  |
| 14 | 3 | - | 5 |  | 408 | 9.9 | 2.40% | A | N |  | N | N | G | N | N | N | N | N | N | ex7/ms |
| 15 | 3 | - | 5.3 |  | 15 | 0.3 | 2.20% | A | L |  | N | G | N | N | N | L | N | L | N |  |
| 16 | 2 | + | 5.3 |  | 352 | 3.7 | 1.10% | A | G |  | N | U | N | G | G | G | U | G | G | ex5/ms |
| 17 | 3 | + | 6 |  | 243 | 9.6 | 3.90% | A | G | p.H1047R | N | L | N | N | N | G | N | G | G | ex5/ms |
| 18 | 3 | - | 7.1 |  | 2035 | 37.9 | 1.90% | A | N |  | N | N | N | G | N | N | N | N | N |  |
| 19 | 2 | - | 8.2 |  | 360 | 4.9 | 1.40% | A | N |  | N | G | N | N | N | N | N | G | G |  |
| 20 | 3 | - | 10.2 |  | 149 | 1 | 0.70% | A | N |  | N | N | A | G | N | N | L | L | N |  |
| 21 | 3 | - | 10.4 |  | 169 | 3.4 | 2.00% | A | N |  | N | N | N | N | N | N | N | N | N |  |
| 22 | 1b | - | 13.2 |  | N. D. | N. D. | N. D. | A | G |  | N | N | N | N | N | N | U | N | N |  |
| 23 | 3 | - | 18.1 |  | 1420 | 19.9 | 1.40% | A | N |  | N | L | G | A | G | L | U | L | L |  |
| 24 | 2 | - | 21.4 |  | 208 | 1.8 | 0.90% | A | N |  | L | G | N | N | N | L | N | N | L | ex7/ms |
| 25 | N.E | MBC | 4.6 | 8/2 | 25 | 0.4 | 1.40% | A | N |  | G | N | G | A | N | N | GU | G | G |  |
| 26 | N.E | MBC | 9.3 |  | 150 | 1.8 | 1.20% | A | N |  | N | N | N | G | N | N | N | N | N |  |
| 27 | N.E | + | 25.4 |  | 204 | 2.7 | 1.30% | A | G |  | G | G | G | A | G | G | G | G | G |  |
| 28 | 1b | - | N. D. |  | N. D. | N. D. | N. D. | A | N |  | U | U | N | N | N | N | N | G | G |  |
|  |  |  |  |  |  |  |  |  |  |  |  |  |  |  |  |  |  |  |  |  |
| HER2+/HR- type (n=18) | | | |  |  |  |  |  |  |  |  |  |  |  |  |  |  |  |  |  |
| 29 | 3 | + | 0.6 | 1/2 | 154 | 4.8 | 3.10% | N | U |  | N | N | G | G | U | L | N | N | N |  |
| 30 | 1a | - | 1.6 |  | 41 | 0.5 | 1.30% | N | G |  | N | G | G | N | N | N | N | G | G |  |
| 31 | 3 | - | 2.3 | 4/2 | 70 | 1.3 | 1.90% | G | U |  | N | U | N | G | G | N | GU | N | N |  |
| 32 | 3 | + | 3.1 |  | 255 | 7.1 | 2.80% | A | N |  | N | N | N | N | N | N | G | N | N |  |
| 33 | 3 | - | 4.3 |  | 333 | 7.4 | 2.20% | A | N |  | N | G | N | G | N | N | N | N | N |  |
| 34 | 3 | + | 7 |  | 776 | 6.3 | 0.80% | A | N |  | N | N | G | G | N | N | L | N | N |  |
| 35 | 2 | + | 7.1 |  | 2119 | 21.4 | 1.00% | A | N |  | N | N | N | A | N | L | G | N | N |  |
| 36 | 3 | - | 7.3 |  | 2003 | 8.5 | 0.40% | A | G | p.H1047L | N | U | N | N | G | U | G | GU | G | ex8/ms |
| 37 | 3 | - | 10.7 |  | 492 | 12.3 | 2.50% | A | A |  | N | N | G | G | N | L | G | N | N |  |
| 38 | N. E. | + | 10.8 |  | 6 | 0 | 0.40% | A | N |  | N | L | G | N | N | N | G | N | N | ex6/ms |
| 39 | 3 | - | 11.1 |  | 3691 | 65.4 | 1.80% | A | N |  | N | N | N | N | N | N | N | N | N |  |
| 40 | N. E. | - | 12.3 |  | 1171 | 6.6 | 0.60% | A | N |  | N | N | N | G | N | N | G | G | G |  |
| 41 | 3 | - | 12.6 |  | 1020 | 23.2 | 2.30% | A | N |  | N | L | N | L | N | N | L | N | N |  |
| 42 | 3 | - | 14 |  | 2907 | 31.7 | 1.10% | A | N |  | N | N | N | N | G | N | G | L | L |  |
| 43 | 3 | - | 15.5 |  | 2108 | 44.5 | 2.10% | A | G |  | N | G | G | G | G | N | G | N | N |  |
| 44 | 2 | - | 16.3 |  | 2785 | 47.6 | 1.70% | A | N | p.H1047R | N | N | N | N | N | L | N | N | N | ex6/ms |
| 45 | 3 | - | 28.2 | Amplify | 743 | 9.2 | 1.20% | A | N |  | N | N | N | N | N | L | N | N | N |  |
| 46 | N. E. | MBC | 20.5 |  | 286 | 4.6 | 1.60% | A | G |  | N | G | G | G | G | GU | G | G | G |  |

HER2+/HR+, HER2-positive/hormone receptor-positive; HER2+/HR-, HER2-positive/hormone receptor-negative; Res., response to neoadjuvant chemotherapy; Rel, relapse; Copy N.,

Genomic *HER2* copy numbers; Wild-type, wild-type *HER2* mRNA, Δ16, Δ16*HER*2 mRNA; %Δ, percentages of Δ16*HER2* mRNA in wild-type *HER2* mRNA; ex 8/ms, missense mutation in exon 8; Genomic copy number: N, a normal copy; G, gain; A, amplification; L, loss; U, uniparental disomy (UPD); GU, gain of one UPD allele; N. E., not evaluated; MBC, metastatic breast cancer at diagnosis; N. D., not done. FISH, 2/4 indicates that 2 HER2 signals and 4 17q centromere signals were shown in the modal clone of cells.
