# Supplementary material for: Alterations of the genes involved in the PI3K and estrogen-receptor pathways influence outcome in human epidermal growth factor receptor 2-positive and hormone receptor-positive breast cancer patients treated with trastuzumab-containing neoadjuvant chemotherapy
Source: BMC Cancer. 2013 May 16;13:241. doi: 10.1186/1471-2407-13-241 (PMC3663661; doi:10.1186/1471-2407-13-241)
Supplement: Additional file 2: Table S2 — RFS and pCR rates for 34 patients with a HR+ or HR- tumor classified by clinical and genetic characteristics; 8 patients with HER2 copy numbers ≤ 2.0 are excluded. [file 1471-2407-13-241-S2.doc]

Table S2. RFS and pCR rates for 34 patients excluding 8 with *HER2* copy numbers < 2.0

|  | Response to neoadjuvant chemotherapy | | | Relapse-free survival | | | |
| --- | --- | --- | --- | --- | --- | --- | --- |
|  | pCR | No pCR | *P*-value | No. of Patients  (No. of events) | 3-year  estimates | 95% CI | *P*-value |
| All patients | 19 | 11 |  | 34 (7) | 0.77 | 0.61-0.94 |  |
| Response to neoadjuvant chemotherapy  pCR (grade 3)  No pCR (grade 0~2) |  |  |  | 19 (3)  11 (2) | 0.90  0.80 | 0.71-1.09  0.60-1.01 | 0.937 |
| Hormone receptors  Positive  Negative | 8  11 | 9  2 | 0.034 | 19 (3)  15 (4) | 0.80  0.74 | 0.59-1.01  0.47-1.00 | 0.653 |
| Wild-type *HER2* mRNA  < 400  > 400 | 8  11 | 5  4 | 0.505 | 15 (5)  19 (2) | 0.60  0.92 | 0.32-0.88  0.76-1.07 | 0.066 |
| ∆*HER2* mRNA  < 4.5  > 4.5 | 5  14 | 5  4 | 0.132 | 13 (3)  19 (4) | 0.71  0.80 | 0.42-0.99  0.60-1.01 | 0.684 |
| Percentages of ∆*HER2* mRNA  < 2.4%  > 2.4% | 15  4 | 9  0 | 0.137 | 28(5)  4 (2) | 0.81  0.50 | 0.65-0.98  -0.01-0.99 | 0.100 |
| *PIK3CA*  Wild-type  Mutated | 17  2 | 10  1 | 0.9 | 31 (6)  3 (1) | 0.80  0.50 | 0.63-0.96  -0.19-1.19 | 0.620 |
| *PIK3CA*  Wild-type + Normal + Loss + UPD Mutated + Gain | 16  4 | 7  3 | 0.542 | 26 (4)  8 (3) | 0.85  0.48 | 0.69-1.01  0.03-0.92 | 0.060 |
| *PTEN*  Normal + Gain + UPD  Loss | 19  0 | 9  2 | 0.054 | 32(7)  2 (0) | 0.75  1.0 | 0.58-0.93  - | NA |
| *INPP4B*  Normal + Gain + UPD  Loss | 16  3 | 11  0 | 0.165 | 30 (5)  4 (2) | 0.84  0.50 | 0.69-0.99  0.01-0.99 | 0.292 |
| *PI3KCA , PTEN, INPP4B*  No aberrations*  Aberrations** | 13  6 | 6  5 | 0.447 | 21 (3)  13 (4) | 0.88  0.63 | 0.72-1.04  0.33-0.92 | 0.248 |
| *DEK*  Normal  Gain | 13  6 | 11  0 | 0.037 | 26 (4)  8(3) | 0.86  0.50 | 0.71-1.01  0.10-0.90 | 0.1079 |
| *FGFR1*  Normal + Loss + UPD  Gain | 16  3 | 6  5 | 0.077 | 25 (5)  9 (2) | 0.75  0.88 | 0.56-0.94  0.65-1.10 | 0.867 |
| *CCND1*  Normal + Loss  Gain + | 10  9 | 8  3 | 0.279 | 21 (3)  13 (4) | 0.81  0.71 | 0.60-1.01  0.43-0.99 | 0.247 |
| *FOXA1*  Normal + UPD  Gain | 14  5 | 10  1 | 0.256 | 26 (5)  8 (2) | 0.81  0.69 | 0.64-0.98  0.32-1.05 | 0.217 |
| *CDH3*  Normal + Loss + UPD  Gain | 18  1 | 10  1 | 0.685 | 31 (4)  3 (3) | 0.87  0 | 0.71-1.01  0 | <0.001 |
| *BIRC5*  Normal + Loss + UPD  Gain | 13  6 | 8  3 | 0.804 | 21 (2)  13 (5) | 0.88  0.55 | 0.71-1.04  0.18-0.92 | 0.007 |
| *MYBL2*  Normal + Loss  Gain | 17  2 | 7  4 | 0.088 | 26 (4)  8 (3) | 0.86  0.34 | 0.71-1.01  -0.17-0.85 | 0.013 |
| *AIB1*  Normal + Loss  Gain | 17  2 | 7  4 | 0.088 | 26 (4)  8 (3) | 0.86  0.34 | 0.71-1.01  -0.17-0.85 | 0.013 |

CI, confidence interval; *No aberrations, wild-type and a normal copy of *PIK3CA*, and a normal copy, gain or UPD of *PTEN* or *INPP4B*; **Aberrations, mutated and/or gain of *PIK3CA*, and loss of *PTEN* or *INPP4B*
