# Supplementary material for: Alterations of the genes involved in the PI3K and estrogen-receptor pathways influence outcome in human epidermal growth factor receptor 2-positive and hormone receptor-positive breast cancer patients treated with trastuzumab-containing neoadjuvant chemotherapy
Source: BMC Cancer. 2013 May 16;13:241. doi: 10.1186/1471-2407-13-241 (PMC3663661; doi:10.1186/1471-2407-13-241)
Supplement: Additional file 3: Table S3 — RFS in patients with a HR+ or HR- tumor classified by clinical and genetic characteristics; 8 patients with HER2 copy numbers ≤ 2.0 are excluded. [file 1471-2407-13-241-S3.doc]

Table S3.

|  | Hormone receptor-positive tumors (n=19) | | | | Hormone receptor-negative tumors (n=15) | | | |
| --- | --- | --- | --- | --- | --- | --- | --- | --- |
|  | No. of Patients  (No. of events) | 3-year  estimates | 95% CI | *P*-value | No. of Patients  (No. of events) | 3-year  estimates | 95% CI | *P*-value |
| All patients | 19 (3) | 0.80 | 0.59-1.01 |  | 15 (4) | 0.74 | 0.47-1.0 |  |
| Response to neoadjuvant CT  pCR (grade 3)  No pCR (grade 0~2) | 10 (2)  9(1) | 0.88  0.83 | 0.65-1.10  0.54-1.13 | 0.596 | 11 (2)  2 (1) | 1.0  0.78 | -  0.50-1.06 | NA |
| Wild-type *HER2* mRNA  < 400  > 400 | 11 (3)  6 (0) | 0.67  1.0 | 0.35-0.98  - | 0.161 | 4 (2)  11 (2) | 0.38  0.86 | -0.19-0.94  0.60-1.12 | 0.127 |
| ∆*HER2* mRNA  < 4.5  > 4.5 | 11 (2)  6 (1) | 0.79  0.80 | 0.52-1.05  0.45-1.15 | 0.762 | 2 (1)  13 (3) | 0  0.81 | 0  0.56-1.05 | NA |
| Percentages of ∆*HER2* mRNA  < 2.4%  > 2.4% | 15 (2)  2 (1) | 0.85  0.50 | 0.66-1.04  -0.19-1.19 | NA | 13 (3)  2 (1) | 0.76  0.50 | 0.47-1.05  -0.19-1.19 | NA |
| *PIK3CA* (exons 9 and 20)  Wild-type  Mutated | 18 (2)  1 (1) | 0.87  0 | 0.70-1.04  0 | NA | 13 (4)  2 (0) | 0.70  1.0 | 0.41-0.99  - | NA |
| *PIK3CA* (exons 9 and 20)  Wild-type + Normal + Loss + UPD  Mutated + Gain | 15 (0)  4 (3) | 1.0  0 | -  0 | <0.001 | 11 (4)  4 (0) | 0.66  1.0 | 0.34-0.98  - | 0.287 |
| *PTEN*  Normal + Gain + UPD  Loss | 17 (3)  2 (0) | 0.77  1.0 | 0.53-1.0  - | NA | 15 (4)  0 | 0.74  1.0 | 0.47-1.0  - | NA |
| *INPP4B*  Normal + Gain + UPD  Loss | 17 (2)  2 (1) | 0.86  0.50 | 0.68-1.04  -0.19-1.19 | NA | 13 (3)  2 (1) | 0.81  0.50 | 0.56-1.05  -0.19-1.19 | NA |
| *PI3KCA , PTEN, INPP4B*  No aberrations*  Aberrations** | 12 (0)  7 (3) | 1.0  0.54 | -  0.14-0.93 | 0.030 | 9 (3)  6 (1) | 0.74  0.75 | 0.43-1.06  0.33-1.17 | 0.519 |
| *DEK*  Normal  Gain | 15 (2)  4 (1) | 0.82  0.67 | 0.59-1.05  0.13-1.20 | 0.427 | 11 (2)  4 (2) | 0.91  0.33 | 0.74-1.08  -0.20-0.87 | 0.142 |
| *FGFR1*  Normal + Loss + UPD  Gain | 14 (2)  5 (1) | 0.81  0.75 | 0.58-1.05  0.33-1.17 | 0.730 | 11 (3)  4 (1) | 0.66  1.0 | 0.34-0.98  - | 0.954 |
| *CCND1*  Normal + Loss  Gain + | 13 (1)  6 (2) | 0.88  0.60 | 0.65-1.10  0.17-1.03 | 0.132 | 8 (2)  7 (2) | 0.70  0.80 | 0.34-1.06  0.45-1.15 | 0.741 |
| *FOXA1*  Normal + UPD  Gain | 15 (1)  4 (2) | 0.90  0.38 | 0.71-1.09  -0.18-0.94 | 0.013 | 11 (4)  4 (0) | 0.71  1.0 | 0.43-0.99  - | 0.525 |
| *CDH3*  Normal + Loss + UPD  Gain | 16 (0)  3 (3) | 1.0  0 | -  0 | <0.001 | 15 (4)  0 | 0.74  - | 0.47-1.0  - | NA |
| *BIRC5*  Normal + Loss + UPD  Gain | 15 (11)  4 (2) | 0.90  0.38 | 0.71-1.09  -0.19-0.94 | 0.013 | 6 (1)  9 (3) | 0.83  0.59 | 0.54-1.13  0.10-1.09 | 0.235 |
| *MYBL2*  Normal + Loss  Gain | 13 (0)  6 (3) | 1.0  0.30 | -  -0.17-0.77 | 0.002 | 13 (4)  2 (0) | 0.73  1.0 | 0.46-0.99  - | NA |
| *AIB1*  Normal + Loss  Gain | 13 (0)  6 (3) | 1.0  0.30 | -  -0.17-0.77 | 0.002 | 13 (4)  2 (0) | 0.73  1.0 | 0.46-0.99  - | NA |

CI, confidence interval; NA, not applicable; *No aberrations, wild-type and a normal copy of *PIK3CA*, a normal copy, gain, or UPD of *PTEN* or *INPP4B*; **Aberrations, mutated and/or gain of *PIK3CA*, and loss of *PTEN* or *INPP4B*
